# Deep learning-based segmentation of abdominal aortic aneurysms and intraluminal thrombus in 3D ultrasound images

**DOI:** 10.1007/s11517-024-03216-7

**Published:** 2024-10-25

**Authors:** Arjet Nievergeld, Bünyamin Çetinkaya, Esther Maas, Marc van Sambeek, Richard Lopata, Navchetan Awasthi

**Affiliations:** 1 PULS/e group, Department of Biomedical Engineering, Eindhoven University of Technology, De Rondom 70, 5612 AP Eindhoven, The Netherlands; 2 Department of Vascular Surgery, Catharina Hospital Eindhoven, Eindhoven, The Netherlands; 3 Faculty of Science, Mathematics and Computer Science, Informatics Institute, University of Amsterdam, Amsterdam, The Netherlands; 4 Department of Biomedical Engineering and Physics, Amsterdam UMC, Amsterdam, The Netherlands

**Keywords:** Abdominal aortic aneurysms, Intraluminal thrombus, Deep learning, Segmentation, 3D + t US, NnU-Net

## Abstract

Ultrasound (US)-based patient-specific rupture risk analysis of abdominal aortic aneurysms (AAAs) has shown promising results. Input for these models is the patient-specific geometry of the AAA. However, segmentation of the intraluminal thrombus (ILT) remains challenging in US images due to the low ILT-blood contrast. This study aims to improve AAA and ILT segmentation in time-resolved three-dimensional (3D + t) US images using a deep learning approach. In this study a “no new net” (nnU-Net) model was trained on 3D + t US data using either US-based or (co-registered) computed tomography (CT)-based annotations. The optimal training strategy for this low-contrast data was determined for a limited dataset. The merit of augmentation was investigated, as well as the inclusion of low-contrast areas. Segmentation results were validated with CT-based geometries as the ground truth. The model trained on CT-based masks showed the best performance in terms of DICE index, Hausdorff distance, and diameter differences, covering a larger part of the AAA. With a higher accuracy and less manual input the model outperforms conventional methods, with a mean Hausdorff distance of 4.4 mm for the vessel and 7.8 mm for the lumen. However, visibility of the lumen-ILT interface remains the limiting factor, necessitating improvements in image acquisition to ensure broader patient inclusion and enable rupture risk assessment of AAAs in the future.

**Graphical abstract:**

**Figure:**
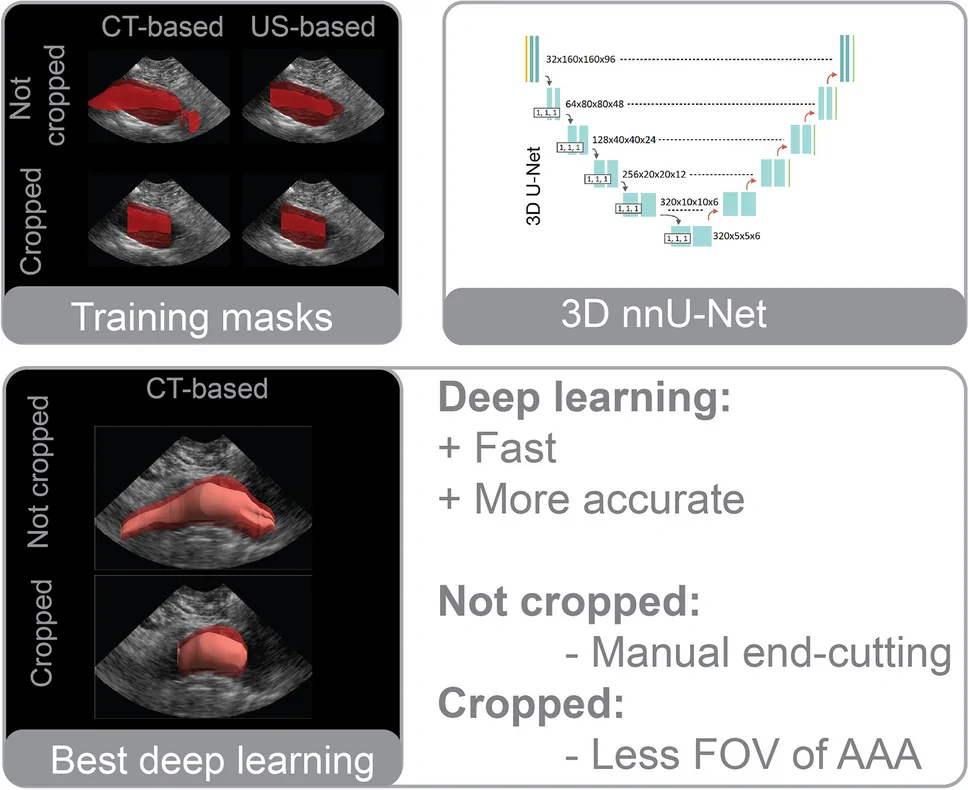

## Introduction

An abdominal aortic aneurysm (AAA), a permanent dilation of the aorta, is an asymptomatic disease, which in case of rupture has a mortality rate of 80% [1]. Close monitoring of AAAs is essential in rupture risk prediction. Currently, the maximum anterior–posterior AAA diameter is used as a criterion for surgical intervention, which is typically monitored using two-dimensional ultrasound (2D US) [2, 3]. Nevertheless, this diameter-based rupture risk prediction lacks adequate specificity to individual patients, as some AAAs rupture at small diameters, while others remain stable at significantly larger diameters [4, 5]. This underscores the necessity for a more personalized approach. Around 75% of all AAAs contain intraluminal thrombus (ILT) [6]. A controversy exists about its exact role on AAA rupture risk. Studies have shown that the presence of an ILT reduces the wall strength due to attenuation of oxygen diffusion towards the vessel wall [7]. Conversely, ILT has a demonstrated cushioning effect, reducing stress transmission towards the wall, lowering the chance of rupture from a mechanical perspective [8].

Model-based rupture risk prediction has been suggested as a biomechanical alternative for 2D diameter monitoring, showing promising results [9–11]. Studies showed that the peak wall stress (PWS) was higher in ruptured AAAs, compared to non-ruptured AAAs [12, 13]. However, AAA rupture is not only dependent on wall stress, but will occur when the wall stress exceeds the wall strength [14]. Vande Geest et al. developed a model to estimate the wall strength based on local ILT thickness, local AAA diameter, family history, and gender [15]. This estimated wall strength could be used in combination with the wall stress to determine the so-called peak wall rupture index (PWRI). When using the PWRI, even higher differences between the ruptured and non-ruptured AAA group were visible, compared to the PWS [10, 14, 16–19]. Singh et al. evaluated the potential value of using the PWRI in AAA classification and found a positive net reclassification index, when using both PWRI and diameter together, compared to solely relying on diameter [20].

To calculate the PWRI, the 3D geometry of the entire AAA including the ILT is required. Most studies calculate the PWRI using computed tomography (CT) [18, 21]. CT allows delineation for both the vessel-ILT and ILT-luminal borders. However, drawbacks are the use of X-ray and nephrotoxic contrast agents. US is a safe, cheap, and easy-to-use alternative, making it the preferred imaging modality for regular follow-ups.

Additionally, the dynamic information in time-resolved three-dimensional (3D + t) US allows for mechanical characterization of the vessel tissue, which can be used to further personalize rupture risk prediction [22]. However, US also has its drawbacks, i.e., a limited field-of-view (FOV) and a relatively poor contrast of the ILT-lumen interface, making it challenging to accurately segment the entire AAA. A previous study already showed the feasibility of segmenting ILT and AAA wall in 3D + t US images using non-deep learning (conventional) segmentation techniques [23]. However, this method lacked robustness and accuracy in areas with low tissue contrast.

Deep learning techniques have emerged as a faster and more powerful tool in medical image segmentation, also for AAAs [24–26]. Maas and Awasthi et al. demonstrated that a “no new net” (nnU-net) deep learning model trained on 3D + t US data enhances the accuracy and robustness of AAA vessel wall segmentation, surpassing conventional segmentation approaches [27]. However, this study did not consider segmentation of the lumen-ILT border.

The main objective of this paper is to develop a deep learning-based model for automatic and adequate segmentation of the AAA, i.e., lumen, ILT, and vessel wall, from clinical 3D + t US images, in an accurate and robust fashion. A nnU-Net model [25] was chosen and its performance was assessed for different learning strategies, considering factors such as data augmentation, cropping low-contrast US regions, and the use of either US masks or annotations obtained from co-registered CT-data. An extensive comparison is made to come to an optimal method for the data given. Obtaining a sufficient amount of ground truth training masks for the lumen-ILT border is challenging compared to the vessel wall, due to poorer echogenicity of the ILT and poor contrast of the lumen-ILT interface. Therefore, this study investigates the use of a multi-modality approach, where co-registered CT-based masks were used for training as an alternative to US-based labels. The added advantage of employing such a multi-modality approach in model training is that these CT-based labels rely on high-contrast images that remain unaffected by clutter or other image artifacts commonly found in US images.

## Materials and methods

### Data acquisition

US images were acquired in the Catharina Hospital Eindhoven as part of an ongoing prospective observational study. The study was approved by the local ethics committee of the Catharina Hospital Eindhoven (reference number: 2014–62 (CZE)), and all patients gave their written informed consent. The time-varying US images were obtained during breath hold using the Philips iU22 and Epiq G7 US system, both equipped with a 3.5 MHz X6-1 probe. All CT angiography (CT-A) scans were acquired for pre-operative planning as a part of the normal clinical workflow.

In total, 233 patients (723 US datasets) were included, with a (partially) visible ILT. From this dataset, 33 US images from different patients had to be excluded, due to segmentation problems with the conventional segmentation method. This resulted in 690 US datasets obtained in 229 different patients. A subgroup of 54 out of 229 patients had one or multiple CT scans acquired within 100 days of US acquisition. These data were co-registered to the US datasets (see Section 2.2) and used for model training and testing. The CT data of three patients had to be excluded due to registration errors, resulting in 78 US datasets of 51 patients.

Both the dataset containing all US data (L) and the subgroup (S) with co-registered CT scans were divided in a training and test group, following a 3:1 ratio. The participant demographics and AAA characteristics for testing and training are given in Table 1. The data obtained with either the iU22 or Epiq systems were evenly distributed over the different groups. It must be noted that the datasets from a single patient were exclusively used in either the testing or training group.

**Table 1:** Participant demographics and AAA characteristics for the testing and training datasets

	Training		Testing	
	CT dataset	US dataset	With CT	Without CT
Participant characteristics:				
No. of participants	38	145	13	14
No. of women (%)	9 (24%)	26 (18%)	2 (15%)	1 (7%)
Median (range) age	78 (72–82)	77 (53–94)	70 (59–87)	73 (66–82)
AAA characteristics:				
Median (IQR) maximum diameter [cm]	5.3 (5.2–5.5)	4.9 (4.5–5.3)	5.3 (5.0–5.4)	3.4 (3.3–3.8)
Median (IQR) AAA center depth [cm]	6 (5.5–7.0)	5.6 (4.9–6.5)	6.1 (5.6–6.8)	6.0 (5.6–7.1)

Qualitative testing was performed by comparing the segmentation results of the different models (see Section 2.3) with CT annotations. For this comparison, a subgroup including the remaining 18 US datasets was available. Finally, the performance of the segmentation was also tested on smaller AAAs (49 datasets acquired in 14 patients). For these cases, typically no CT data are available; hence, a (qualitative) visual inspection was performed.

### Data preprocessing

All datasets were pseudonymized and processed in MATLAB (R2021a, MathWorks, Natick, MA, USA). US-based segmentations (and subsequent binary masks) were generated through a semi-automatic segmentation approach, previously published by our group [23]. The US images were filtered using a Euclidean Shortening Flow filter to reduce noise and artifacts. Both the lumen-ILT and the thrombus-wall interface were detected in two steps. A star-Kalman algorithm was used to find initial contours, which were refined in a second step by applying a conventional 3D snake algorithm. The method was implemented in MATLAB. For further details, see Nievergeld et al. [23].

CT scans were semi-automatically segmented using Hemodyn (Philips Medical Systems, Best, The Netherlands, and the Eindhoven University of Technology, TU/e) [28, 29]. The resulting CT-geometries were co-registered onto the US-based geometries using the iterative closest point (ICP) algorithm. Both the co-registered CT-geometries and US-based geometries were transformed into masks. All US images and masks were resampled to a 0.5 × 0.5 × 0.5 mm Cartesian grid.

### Models and training

The nnU-net used in this study is a powerful neural network for accurate segmentation of organs in 3D medical imaging data [25, 27]. The nnU-net was developed and validated on datasets provided by the Medical Segmentation Decathlon. The method is able to perform automated configuration on arbitrary new datasets, covering the entire segmentation pipeline without any manual decisions. The general applicability of nnU-Net was demonstrated in additional datasets, with results on numerous segmentation tasks covering an unprecedented diversity of target structures, image types, and image properties [25, 27]. The method is open-source and can be trained easily to produce state-of-the-art segmentations. The automated configuration process is fast and requires minimal computational resources. Additionally, it is data efficient, encoding design choices based on a large and diverse data pool that serves as a strong basis for application to datasets with limited training data [25, 27].

After the conversion of the datasets to the decathlon format, the nnU-Net’s network architecture is optimized for the specific dataset during the planning phase [25]. The configuration for the segmentation pipelines incudes the fixed parameters, rule-based parameters, and empirical parameters which depend on the dataset [25]. The models are trained utilizing a batch size of 2, learning rate of 1e-2, and a stochastic gradient decent optimizer for 1000 epochs. The models using the nnU-net library are run on Snellius, which is the Dutch National supercomputer. The GPUs in these nodes are 4 × NVIDIA A100, with 40 GB of HMB2 memory and 5 active memory stacks per GPU.

**Fig. 1:**
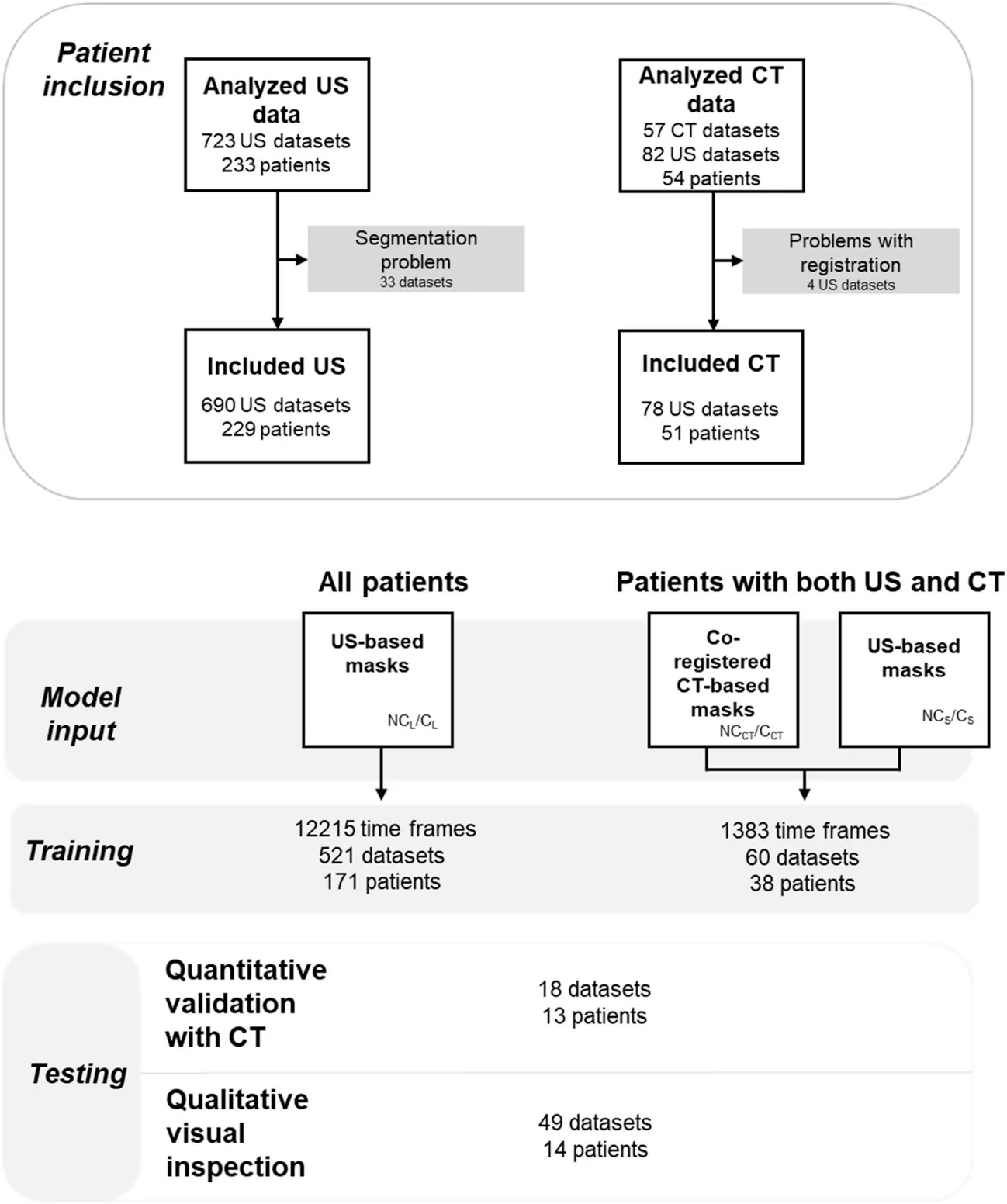
Flowchart of CT and US datasets, illustrating dataset exclusions and various model inputs: L^C^/L represents a large US dataset including all patients with a visible ILT in the US data, S_CT_/S^C^_CT_ refers to the subgroup of patients having a CT, trained with CT-annotations, and S^C^/S refers to the same subgroup of patients with an available CT scan trained with US-based masks. Superscript C denotes cropped masks were used, and no superscript denotes the full FOV masks were used

Here, a 3D full-resolution (FullRes) nnU-Net architecture [25] was used, trained with two different input masks: the US-based labels and co-registered CT-based labels. The performance of the model trained with US-based segmentations was evaluated on:

- The full US dataset including all patients (L).
- A subgroup dataset (S) of patients for which a co-registered CT was available. This subgroup was created to evaluate the impact of training models with either co-registered CT-based masks (S_CT_) or US-based masks (S) models with an identical number of input datasets (Fig. 1).

**Fig. 2:**
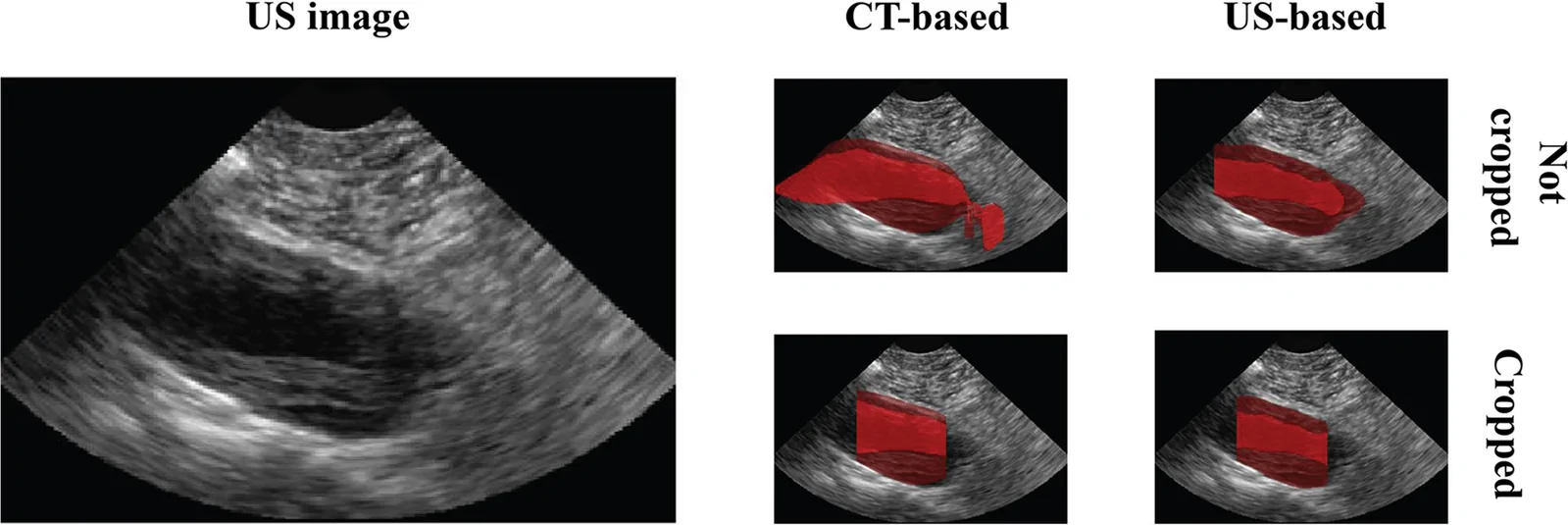
Example patient dataset, with the ultrasound image (left), and corresponding co-registered CT-based (middle) and ultrasound-based mask (right) for both the non-cropped and the cropped configurations. The lumen is visualized in bright red, and the intraluminal thrombus in dark red

The visibility of the AAA in the 3D (pyramidal) US volumes decreases for larger steering angles. Hence, training was performed using the entire FOV and for a limited FOV, where the low-contrast regions in the US images were omitted from the analysis by cropping the masks accordingly. Therefore, all three models (L, S, and S_CT_) were trained for both the full FOV, or non-cropped dataset (no superscript), and the cropped dataset (superscript C), resulting in six different deep learning models (Fig. 1). This cropping of FOV was performed upon visual inspection, and such that the visible parts of the AAA and ILT were included in the binary masks. Figure 2 provides an illustrative example of a patient, showing both the full and cropped versions of these CT-based and US-based input masks.

Finally, all six models were trained with and without data augmentation. Augmentation is typically used in deep learning to increase the variety of the training dataset. The data augmentation included mirroring, rotation and scaling, addition of Gaussian noise, Gaussian blur, variations in brightness and contrast, simulation of lower image qualities, and gamma augmentation [25, 27].

### Post-processing

The masks generated by the deep learning models were converted into 3D contours. These contours were truncated at points where the overlap between two consecutive slices fell below 50% or when the radius was less than 5 mm (therefore indicating incorrect segmentation).

### Evaluation of segmentations and statistical evaluation

The performance of all 12 models, the result of the different training strategies discussed, was compared and verified with CT data for all US datasets that were available (*N* = 18, acquired in 13 patients). The similarity was determined by calculating the DICE similarity index ($\mathrm{DICE}$), one of the most common measures for region overlap in medical image analysis [30], and the 95^th^ percentile Hausdorff distance (${\mathrm{HD}}_{95}$), which is a quantitative measure for the distance between the segmentations, robust to outliers [31].

1 $$\mathrm{DICE}=\frac{2(A\cap B)}{A+B}$$

With A and B the set of pixels in the ground truth and prediction, respectively. The ${\mathrm{HD}}_{95}$ was calculated as the maximum of the 95^th^ percentile directed distances ${d}_{95}\left(c,U\right)$ and ${d}_{95}\left(u,C\right)$. (Eqs. (2) and (3))

2 $${d}_{95}\left(c,U\right)={{}_{\hspace{0.17em}}{}^{95}K}_{a\epsilon A}^{th}d\left(c,U\right)$$

3 $${\mathrm{HD}}_{95}=max\left({d}_{95}\left(c,U\right),{d}_{95}\left(u,C\right)\right)$$

with $d\left(c,U\right)$ is the distance from a point c within the CT geometry to the closest point U in the US-geometry, and vice versa for $\mathrm{d}\left(u,C\right)$.

Initially, the performance of the different models was analyzed quantitatively in the overlapping region, i.e., the region where segmentation results were available for all approaches. Possible statistical differences were analyzed using the Wilcoxon signed-rank test.

Based on the $\mathrm{DICE}$ and ${\mathrm{HD}}_{95}$ performance, the three best-performing models were selected. These models underwent a more comprehensive analysis, focusing on model performance in low-contrast regions compared to the conventional segmentations. Slice by slice, the maximum Hausdorff distance (${HD}_{100}$), $\mathrm{DICE}$, and the difference in local diameter perpendicular to the centerline [32] between the CT- and US-based segmentation were computed. The latter is a geometrical parameter, which is more robust to registration errors due to the lower number of degrees of freedom. The segmentation was divided in three regions. The first region corresponds to the US slices in which a conventional segmentation is available; the second region encompasses US slices where no conventional segmentation is available due to limited contrast in the US image but still shows a visible AAA; in the third region, the AAA cannot be depicted from the US data. In these three regions, the mean ${\mathrm{HD}}_{100}$ and $\mathrm{DICE}$ and the root mean square error (RMSE) of the diameter difference between US and CT were calculated.

## Results

### Accuracy in overlapping region

Before discussing the segmentation results, we want to note that the use of data augmentation either enhances the results or shows no significant impact. Results are available in Fig. 7 of Appendix. The segmentation resulting from the model with the CT-based dataset shows a significant improvement in DICE for the model with data augmentation, compared to the models without data augmentation. For the models trained with the full US dataset, no significant difference was observed (*p* > 0.05). Hence, only models with data augmentation will be considered and discussed in the next sections.

**Fig. 3:**
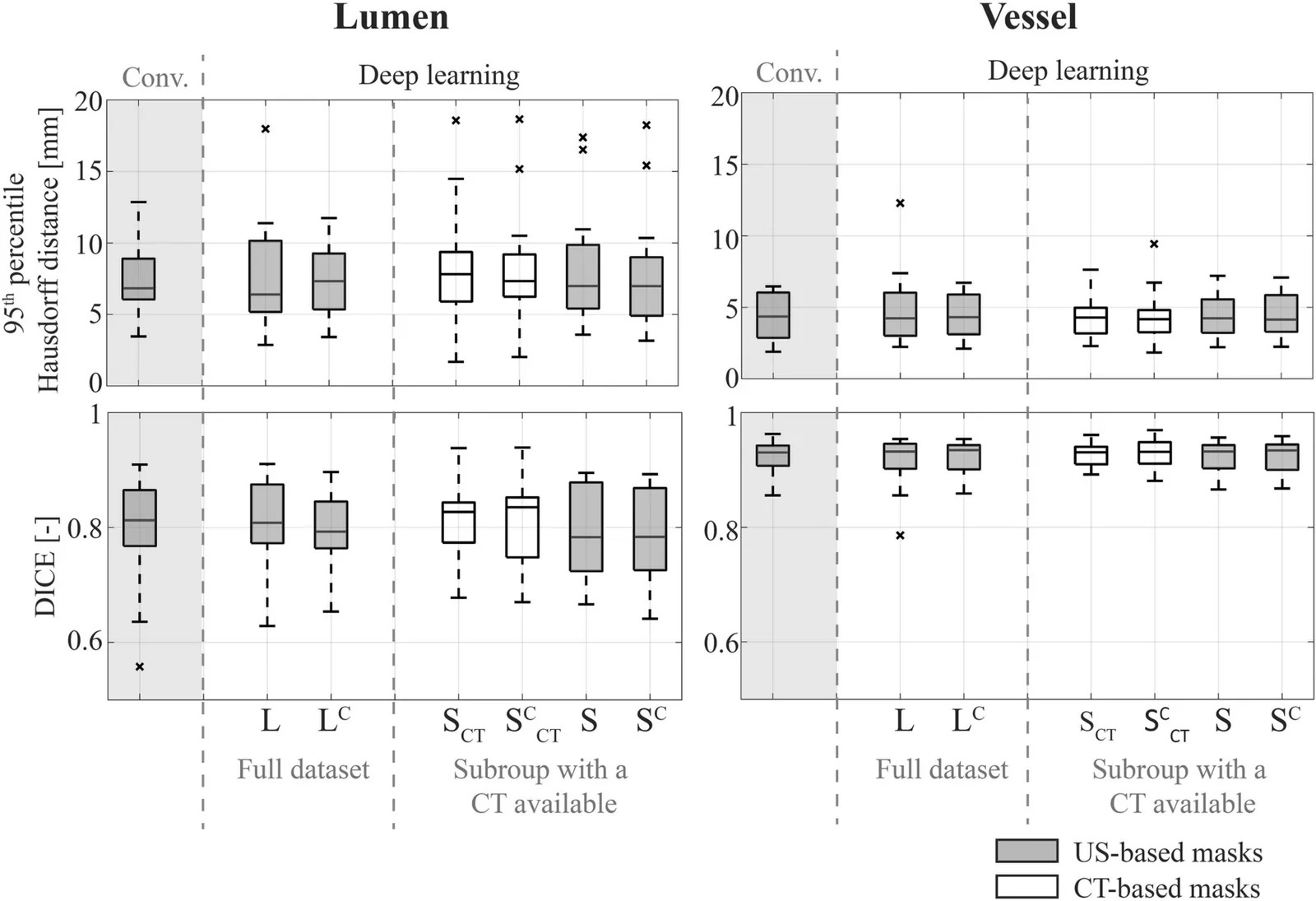
DICE score and the 95^th^ percentile Hausdorff distance for both the lumen and inner vessel wall segmentation. Box-and-whiskers plots show the results of the conventional (Conv.) segmentation method and deep learning segmentation method. L^C^/L represents a large US dataset including all patients with a visible ILT in the US data, S_CT_/S^C^_CT_ refers to the subgroup of patients having a CT, trained with CT annotations, and S^C^/S refers to the same subgroup of patients with an available CT scan trained with US-based masks. Superscript C denotes cropped masks were used, and no superscript denotes the full FOV masks were used

Figure 3 illustrates that vessel wall segmentation achieves higher accuracy than the lumen segmentation, as shown by the higher $\mathrm{DICE}$ (*p* < 0.001) and the lower ${\mathrm{HD}}_{95}$ (*p* < 0.001) for all models, which is expected considering the better echo contrast. The $\mathrm{DICE}$ and ${\mathrm{HD}}_{95}$ between the US-based and CT-based masks revealed no statistically significant differences for all models (*p* > 0.05), for both the vessel wall and lumen. Furthermore, minor performance differences were observed for the vessel wall segmentation, as indicated by a consistent median $\mathrm{DICE}$ of approximately 0.93 across all models.

Although not significant, notable differences were seen in the performance of lumen segmentation, i.e., the lumen-ILT boundary. The median $\mathrm{DICE}$ of the CT-trained models surpassed that of corresponding US-trained models (median $\mathrm{DICE}$: 0.83 and 0.84 for S_CT_ and S^C^_CT_, and 0.81 and 0.79 for L and L^C^). The median $\mathrm{DICE}$ and ${\mathrm{HD}}_{95}$ of the cropped CT-trained model (S^C^_CT_) were slightly better compared full FOV model (S_CT_). Both CT-trained models were included in further analysis since the resulting FOV segmentations were significantly longer (*p* < 0.001), with a median of 9.7 cm, compared to a median length of 6.2 cm for the cropped CT-trained model.

For the US-based models, the cropped model trained with the full dataset (L^C^) shows the highest DICE (median: 0.81) and lowest HD_95_ (median: 6.4 mm), revealing the added value of more training data and not cropping the masks of the training set. In fact, the HD_95_ was even 0.9 mm lower than that found in the model trained on CT-based masks. Therefore, the US-model trained with the full dataset was also included in the remainder of our analysis.

### Accuracy along the AAA axis

**Fig. 4:**
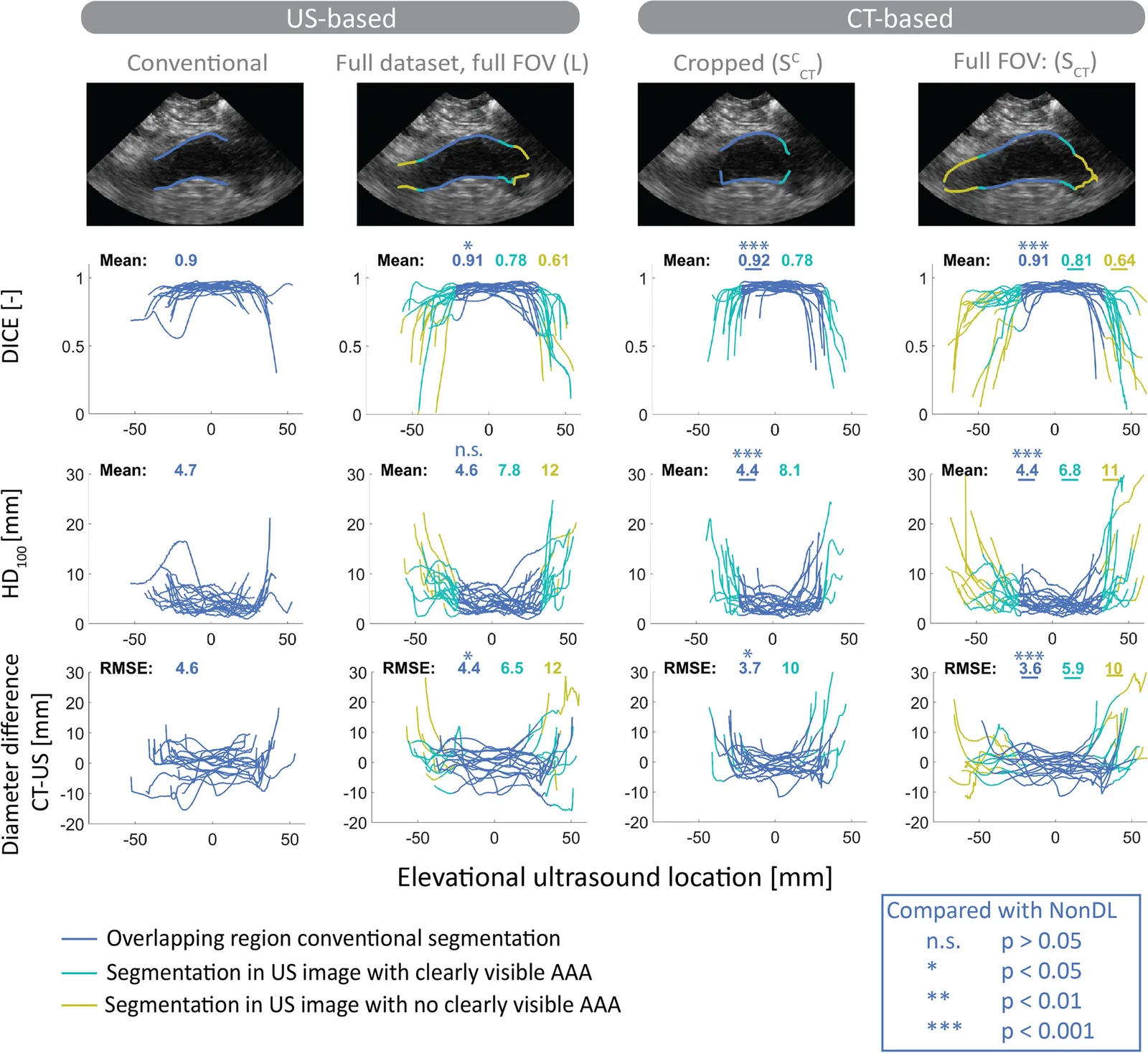
Vessel segmentation results of 4 different models, comparing the DICE score, Hausdorff distance (HD_100_), and diameter difference between US and CT along the elevational ultrasound direction (= vessel axis), with 0 mm equal to the center of the ultrasound image. The different colors represent the different regions of the segmentations, as indicated in the legend. For every region, the mean DICE, HD_100_, and RMSE for diameter difference were calculated, with the underlined value showing the best model for that metric. The US image with an example segmentation of that specific model is given in the top row

Figure 4 shows the vessel wall segmentation $\mathrm{DICE}$, ${\mathrm{HD}}_{100}$, and diameter difference between US and CT along the elevational US coordinate which aligns with the axis of the AAA. Figure 4 reveals that shape of the DICE curve along the vessel’s axis (i.e., elevational direction) has a convex shape. The $\mathrm{DICE}$ increases, followed by a plateau before decreasing in value again). The ${\mathrm{HD}}_{100}$ and diameter difference between US and CT are the opposite, showing a concave curve for all models, with an optimum around the middle of the US image as expected, conforming that the segmentation becomes less reliable in the outer regions of the US volume. In the blue (overlapping) region, the mean $\mathrm{DICE}$ (range 0.90–0.92) and ${\mathrm{HD}}_{100}$ (range 4.4–4.7 mm) are comparable for all models. The diameter differences for both CT-trained deep learning models reveal the best results, with a RMSE of 3.6–3.7 mm, compared to 4.6 mm of the conventional model. The accuracy of the segmentation beyond that of the conventional model (yellow and green region) is highest for the full FOV CT-trained (S_CT_) model, indicated by all metrics in both regions.

**Fig. 5:**
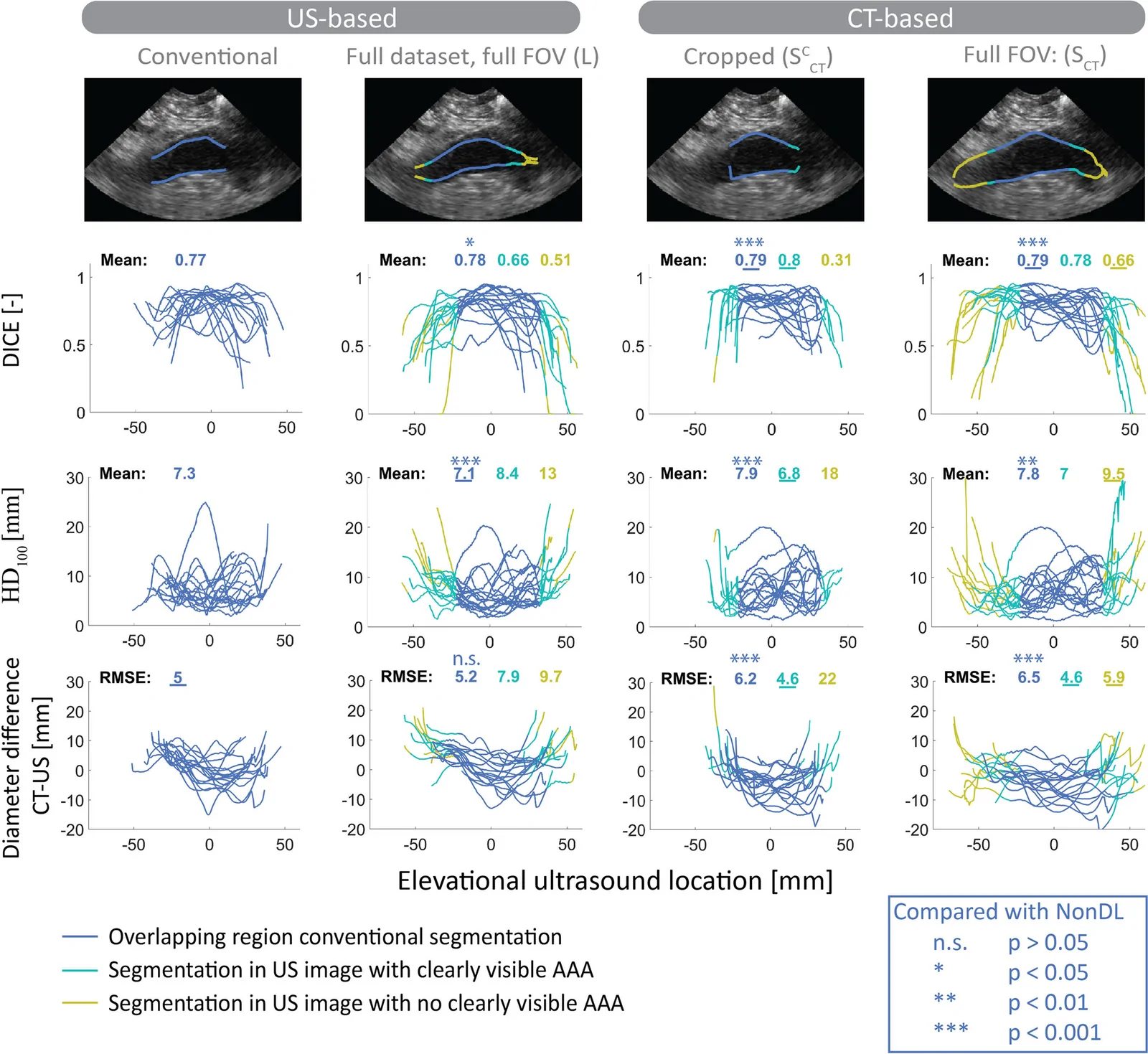
Lumen segmentation results of three models trained with different learning strategies, and the non-deep learning segmentation, comparing the DICE score, Hausdorff distance (HD_100_), and diameter difference between US and CT (along the elevational ultrasound direction, with 0 mm equal to the center of the 3D US image. The different colors represent the different regions of the segmentations. For every region, the mean DICE, HD_100_, and RMSE for diameter difference were calculated, with the underlined value showing the best value. An US image with segmentation generated with each specific model is given in the top row

Figure 5 shows the segmentation results of the lumen. Interestingly, this figure shows, that the HD_100_, and diameter for the CT-trained models are better outside the working range of the conventional model (green), than the ones in the overlapping region (blue), in which generally speaking an overestimation of the US diameter was observed. Both the conventional and full dataset US models also performed significantly better in this mid-region. Conversely, in that same region, the $\mathrm{DICE}$ of the CT-trained model output is significantly better, compared to conventional segmentation. Outside this mid-region, the CT-trained models are performing better for all three metrics, compared to the L model. In the region of the US image in which no AAA is visible (yellow), the CT-trained model has the best results for all three metrics, although the $\mathrm{DICE}$ and ${\mathrm{HD}}_{100}$ show that the performance is not as accurate (mean of 0.66 and 9.5 mm, respectively).

The CT-trained models, showed overall, the best results, based on DICE, HD_95_, and diameter difference, prompting further examination through visual inspection.

**Fig. 6:**
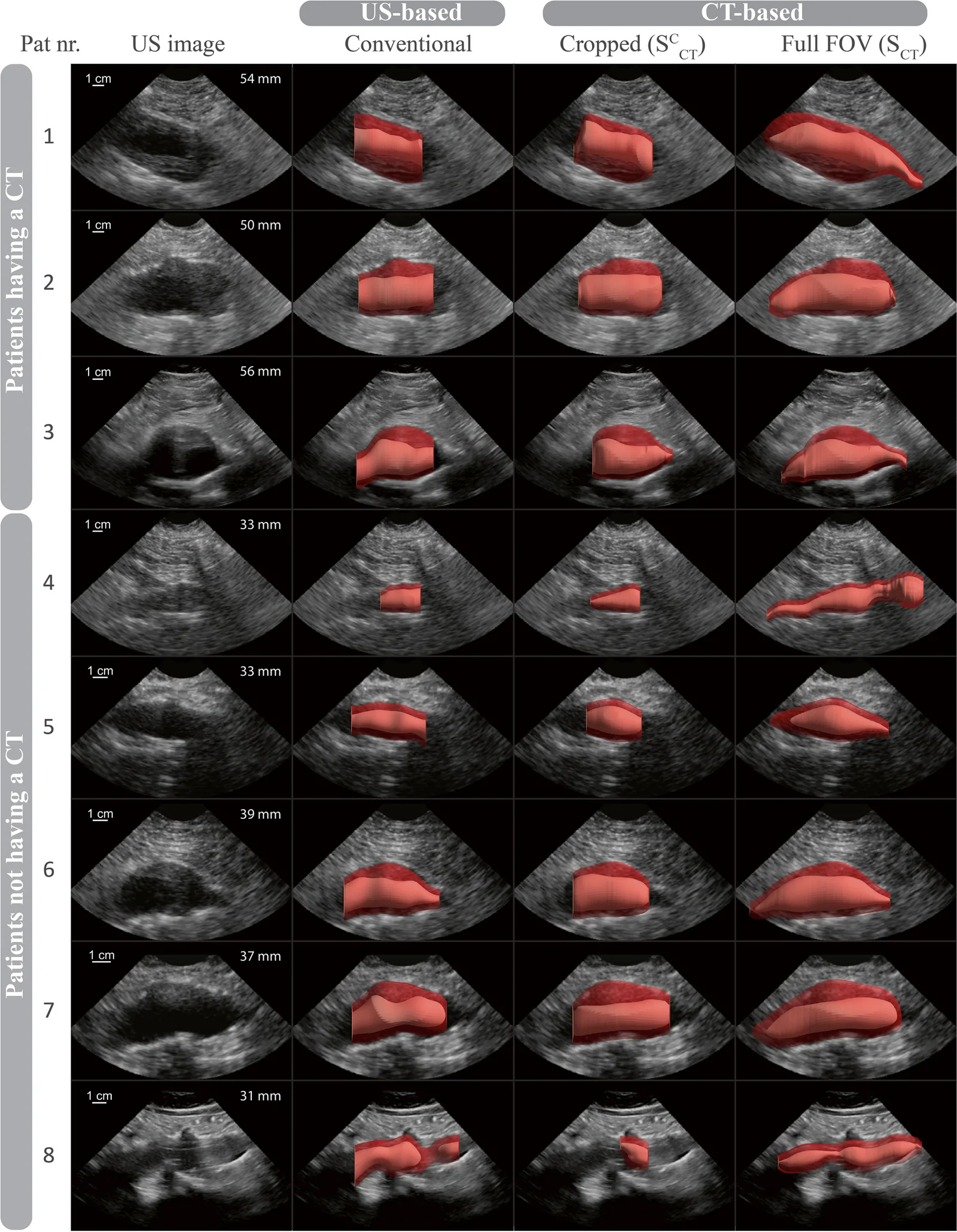
Examples of different patients with the US images (first column), the conventional model (second column), and the CT-based cutoff, and non-cutoff models (third and fourth column). With the lumen rendered in light red, and the intraluminal thrombus in dark red. Examples are given for patients for which a co-registered CT was available (top three rows) and for patients with only US datasets (bottom five rows)

**Fig. 7:**
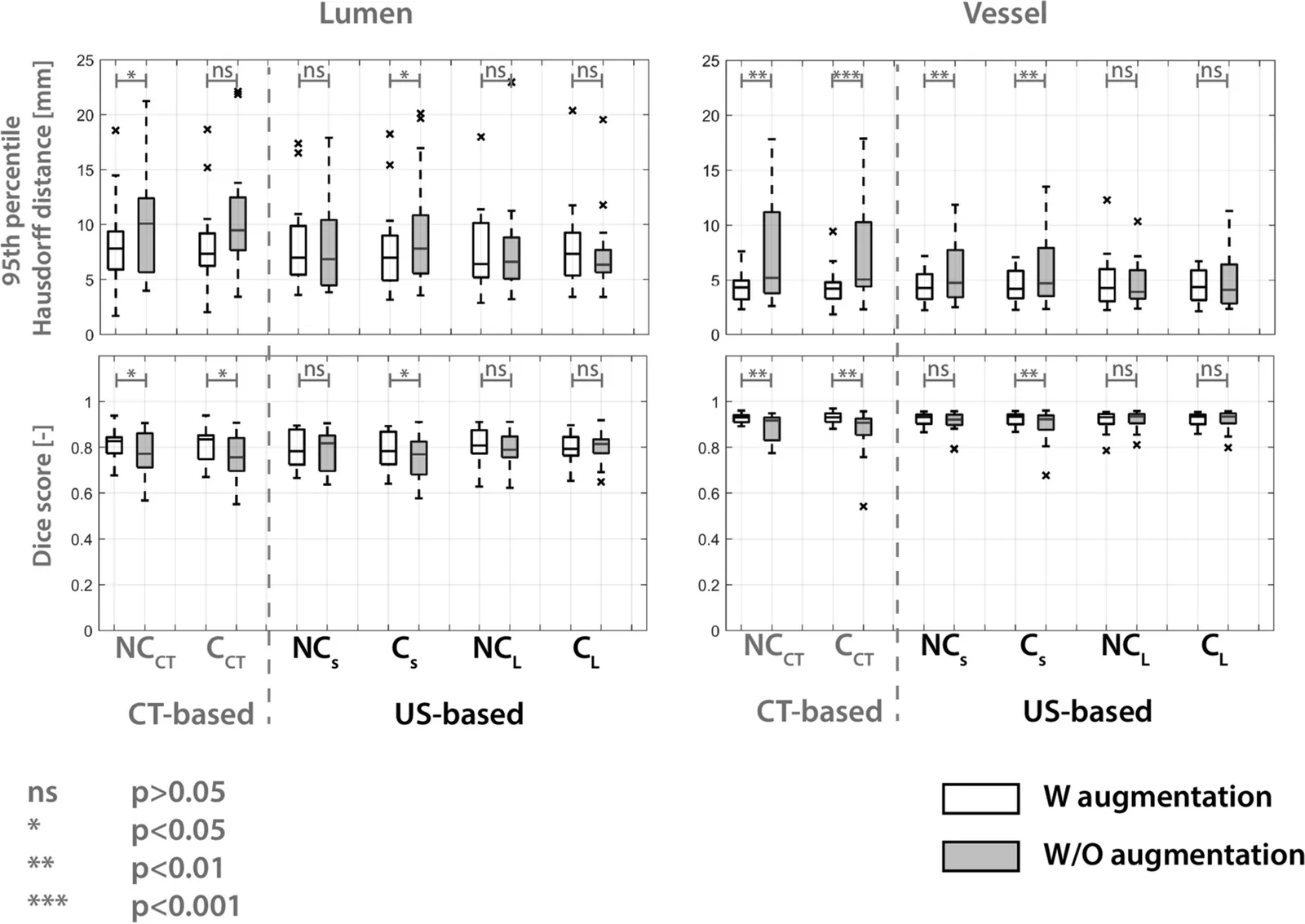
DICE score and 95^th^ percentile Hausdorff Distance for both the lumen and inner vessel wall, comparing the deep learning models with data augmentation (white), and without data augmentation (gray). The models are trained with either the full dataset (L) or a subgroup (S) of patients having a co-registered CT available. The subgroup of patients was used to train the model either with CT-based masks (subscript CT) or with US-based masks (no subscript). All those three models were either trained with the full FOV data (no superscript) or with the cropped data (superscript C)

Figure 6 shows the segmentation outcomes of both the conventional model and the CT-trained models (full FOV and cropped). Generally, the full FOV CT-trained model is able to segment a larger part of the AAA in the US data, even in the low-contrast regions, except for patient 4 where the segmentation appears unrealistic and overly elongated compared to the more realistic, yet shorter segmentation by the cropped model. In patient 1, the full FOV CT-trained model extends the geometry even beyond the US image boundary, which is unexpected. The segmentation of patient 8 of the cropped CT-trained model seems to be incomplete and relatively short. The segmentations of the both CT-trained models are comparable to the conventional model in the overlapping regions for all patients. Both models correctly segment the AAA for both the datasets with and those without CT scans. This shows generalizability of the models towards cases with smaller AAA diameters, see, e.g., patients 4 and 5.

## Discussion

Automatically segmenting the ILT and vessel wall of AAAs in 3D + t US remains a challenge. In this study, the feasibility of deep learning-based segmentation of different regions was demonstrated. Specifically, the network was trained with different inputs, i.e., segmentation masks obtained from CT and US data. The $\mathrm{DICE}$ and ${\mathrm{HD}}_{95}$ for the vessel wall showed better results than the lumen, which is in line with our previous study using conventional segmentation [23]. Generally speaking, the best-performing deep learning models were those trained with CT-based labels, in most cases, outperforming the conventional models. Only in the mid-region of the lumen, some conflicting results were seen for the $\mathrm{DICE}$, ${\mathrm{HD}}_{100}$, and diameter differences. The $\mathrm{DICE}$ for the deep learning CT-based models was higher, whereas the ${\mathrm{HD}}_{100}$ and diameter difference in the US-based conventional model was lower. Outside the overlapping region, the CT-based models were outperforming the US-based models for both the lumen-ILT interface and vessel wall. Overall, the vessel and lumen-ILT interface segmentation of the fully automatic deep learning CT-based segmentations were better than the laborious semi-automatic conventional segmentations. Since the CT data were acquired for pre-operative planning and contained mainly large AAAs, the models were also tested on patients with a small(er) AAA diameter. Visual inspection revealed that despite CT-based training, possibly biased by the use of mostly large AAA diameters, the model still demonstrates proficient performance when applied to these US datasets.

For proper evaluation of AAAs, it is preferred to obtain the longest segmentation possible from the available US. The CT-based model output (using the full FOV in the US images) resulted in segmentations with a median length of 9.7 cm, in contrast to the 6.2-cm length of the cropped CT-based model and conventional results. As depicted in Fig. 6, the deep learning model also captures a larger portion of the inlet and outlet in these low-contrast regions, which is promising for future clinical application. The contours align with the darker regions in the US image, most likely corresponding to the lumen. Nonetheless, the segmentation errors increased near the edges of the US image. The models based on the full field-of-view datasets likely necessitate manual removal of erroneous segments, a practice not preferable for clinical acceptance or large-scale patient studies. Expanding FOV with novel US techniques can overcome these issues [29, 33].

The main limitation of this study is the small dataset of patients with co-registered CT data. Although the model trained with CT was performing best, it only consisted of 60 3D + t US images, amounting to a total of 1383 time frames. A larger dataset with more patients could improve its performance by introducing more variation in geometry and image quality. This was already observed in this study when using both small and large datasets, albeit with marginal differences (median $\mathrm{DICE}$ is 0.78–0.78 and 0.79–0.81, respectively). The registration errors between US and CT scans within the training dataset, compounded by segmentation errors in both modalities (US and CT), are also a limiting factor which likely influenced the outcomes of CT-based training and validation results.

Data augmentation significantly improved the results for this small dataset. Although augmentation strategies in line with previously documented studies [34–36] were used, they may not always apply to US imaging. Speckle patterns in US images are highly directional, which makes rotated or scaled US images less realistic. Ideally, augmentation methods that mimic the image features and artifacts from the transducer and the medium, e.g., attenuation, acoustic shadowing, or clutter should be employed to expand dataset size and variability. One potential approach to generate images with realistic speckle patterns is through the utilization of generative adversarial networks [37, 38]. However, incorporating these image artifacts was beyond the scope of this study.

The ultimate use of the outcome of this study is to perform patient-specific rupture risk estimation, i.e., US-based PWRI calculation [13, 14, 16, 39], offering a safe and cost-effective approach for patient-specific monitoring of AAA patients. However, an ongoing challenge is of course the poor visibility of ILT in US images in general, mainly due to its hypoechoic character [40]. Consequently, a group of participating patients had to be excluded for this reason. Hence, for use in clinical practice, some major steps need to be taken in improving ILT visibility in US data. Color Doppler imaging and contrast-enhanced US imaging might help to further improve visibility of the ILT [41, 42], although the use of contrast agents would result in a minimally invasive procedure which is not desirable in AAA follow-up. New developments such as multi-perspective imaging [43] have the potential to improve the echogenicity of ILT and thus segmentation performance and model accuracy.

## Conclusion

This study showed that a deep learning approach, trained with co-registered CT data, allows for robust and accurate segmentation of the AAA wall, lumen, and ILT, from 3D + t US images, for a larger part of the FOV. This fast segmentation approach could be used to assess the entire geometry of the AAA in large groups of patients, providing geometrical data on the ILT, thereby opening the possibilities for large-scale patient studies on AAA development and PWRI [39]. To this end, the use of a non-invasive, low-imaging modality will bring personalized treatment of AAAs a step closer to clinical application.

## Appendix

### Glossary of terms

**Table:**

Symbol	Name	Definition	Units
HD_95_	95^th^ percentile Hausdorff distance	Metric giving the 95^th^ percentile maximal distance between two segmentations.	[mm]
HD_100_	Maximum (100^th^ percentile) Hausdorff distance	Metric giving the maximum distance between two segmentations.	[mm]
DICE	DICE index	Metric to determine the volume overlap between two segmentations.	[-]
*A*	Pixels in ground truth	Number of pixels in ground truth.	[px]
*B*	Pixels in prediction	Number of pixels in prediction.	[px]
*d*(*c,U*)	Directed distance	Directed distance from a point c in the CTgeometry to the closest point *U* in the USgeometry.	[mm]
*d*(*u,C*)	Directed distance	Directed distance from a point *u* in the USgeometry to the closest point *C* in the CTgeometry.	[mm]
*d*_*xx*_(*c,U*)	Directed xx^th^ percentile distance	xx^th^ percentile from the directed distance of all points.	[mm]
*d*_*xx*_(*u,C*)	Directed xx^th^ percentile distance	xx^th^ percentile from the directed distance of all points.	[mm]
L		Deep learning model trained with ultrasound segmentations of the full dataset (L).	
S		Deep learning model trained with ultrasound segmentations of a sub group (S) having coregistered CT data available.	
S_CT_		Deep learning model trained with the actual CT (_CT_) segmentations of the sub group (S).	
